# Primary aneurysmal bone cyst of coronoid process

**DOI:** 10.1186/1472-6815-6-4

**Published:** 2006-03-14

**Authors:** Amit Goyal, Isha Tyagi, Rajan Syal, Tanu Agrawal, Manoj Jain

**Affiliations:** 1 Neuro-otology Unit, Department of Neuro-surgery, Sanjay Gandhi Post Graduate Institute of Medical Sciences, Raibareily Road, Lucknow (Uttar Pradesh) – 226 014, India; 2 Department of Pathology, Sanjay Gandhi Post Graduate Institute of Medical Sciences, Raibareily Road, Lucknow (Uttar Pradesh) – 226 014, India

## Abstract

**Background:**

Aneurysmal bone cysts are relatively uncommon in the facial skeleton. These usually affect the mandible but origin from the coronoid process is even rarer. To the best of our knowledge, this is the first reported case of a coronoid process aneurysmal bone cyst presenting as temporal fossa swelling.

**Case presentation:**

A 17 year old boy presented with a progressively increasing swelling in the left temporal region developed over the previous 8 months. An expansile lytic cystic lesion originating from the coronoid process of the left mandible and extending into the infratemporal and temporal fossa regions was found on CT scan. It was removed by a superior approach to the infratemporal fossa.

**Conclusion:**

Aneurysmal bone cyst of the coronoid process can attain enormous dimensions until the temporal region is also involved. A superior approach to the infratemporal fossa is a reasonable approach for such cases, providing wide exposure and access to all parts of the lesion and ensuring better control and complete excision.

## Background

Aneurysmal bone cyst (ABC) usually affects long bones of the body and its involvement of facial bones is relatively rare. Mandible is usual site of involvement in facial skeleton but usually it originates from the region of body and ramus (in the region of molars) [1,2]. At few occasions, it is reported to be derived from the condyle also [3-5]. Involvement of coronoid process is quite rare.

There are few reports found in the literature describing involvement of coronoid process by ABC [6-8]. Mainly coronoid process was involved in one case [6], while ramus was also involved in the other one [7], and extensive involvement of ramus and condyle was also evident in one case [8]. Infratemporal fossa was involved in all these cases. They presented as cheek swelling or swelling in zygomatic region.

We report a case of ABC involving only coronoid process with infratemporal and temporal fossa extension. It presented to us as temporal fossa mass.

It adds one more aspect in diversity of clinical and biological behavior of ABC in maxillofacial region.

## Case presentation

### Case report

A 17 years male patient presented to us with the complaint of swelling in the left side temple since 8 months. There was mild pain on pressing the swelling and on opening of mouth. Swelling was progressively increasing in size. There was no other complaint.

On examination, there was about 3 × 3 cm bony swelling in the left temporal fossa with facial asymmetry due to lateral bowing of left zygoma. It was immobile, slightly tender with egg shell crackling on pressure. There was no murmur or bruit on auscultation. There was no restriction to mouth opening. Lower part of the swelling could be palpated on digital palpation through mouth along the superior part of anterior border of ramus of left mandible.

On aspiration, brownish serous fluid came out. It contained mainly red blood cells.

**Figure 1:**
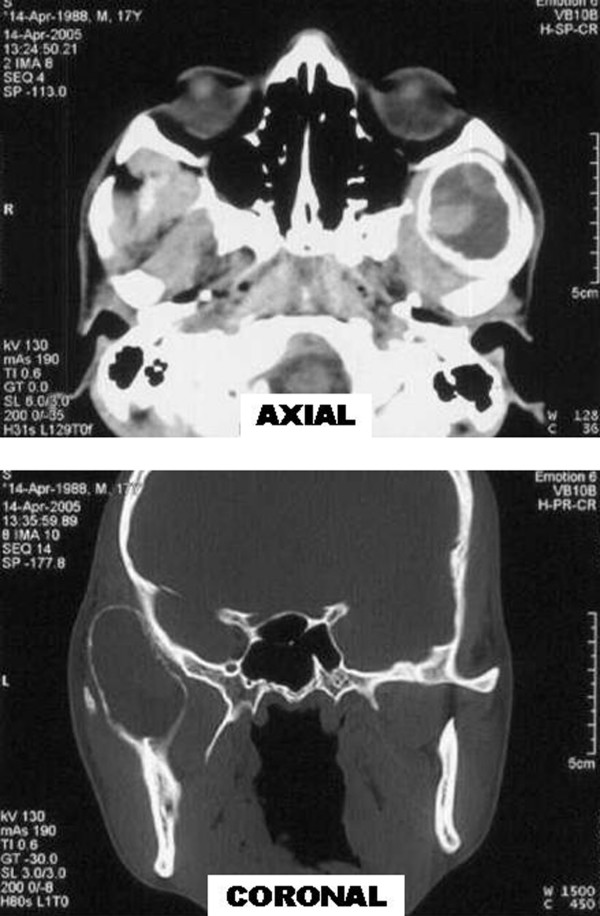
Axial and coronal sections of CT scan showing rounded expansile lesion of bone with cortical thinning, mostly occupied by hypodense fluid with scattered areas of hyperdensity with in it.

CT scan of head and face (Figure 1) revealed rounded expansile lesion of bone with cortical thinning and few areas of cortical erosion. Lesion was originating from coronoid process of left mandible and was extending into the left infratemporal and temporal fossa. Lesion was mostly occupied by hypodense fluid with scattered areas of hyperdensity within it suggestive of haemorrhage.

**Figure 2:**
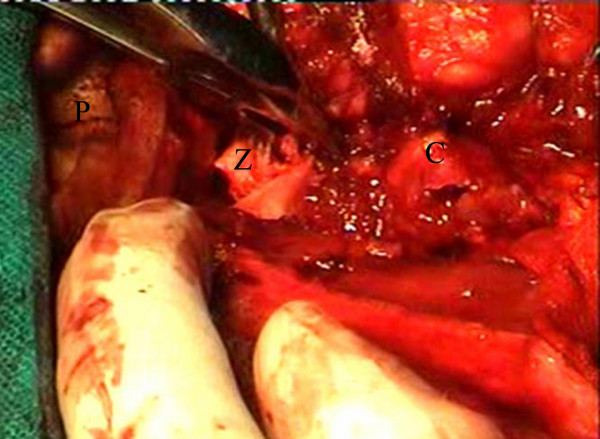
Peroperative photograph showing a thin walled cyst with brownish serous fluid coming out of it. (P = pinna, Z = arch of zygoma, C = aneurysmal bone cyst)

This cystic mass was removed by superior approach to infratemporal fossa. A "Question mark" shaped incision was given within the hair line, starting 5 cm posterosuperior to lateral end of left supraorbital ridge, curving superiorly and posteriorly and coming down vertically to preauricular area. We went directly up to temporalis fascia and incised it. Then we dissected in the plane deep to it. There was a large cyst with papery thin brownish walls filled with brownish serous fluid (Figure 2). Arch of zygoma was cut at anterior and posterior ends. It was then retracted laterally and downwards to expose the infratemporal part of the cyst as done in Fisch D1 approach. Cyst was removed by following it downwards. Cyst was removed completely. The coronoid process was drilled with diamond burr to minimize the chances of recurrence.

**Figure 3:**
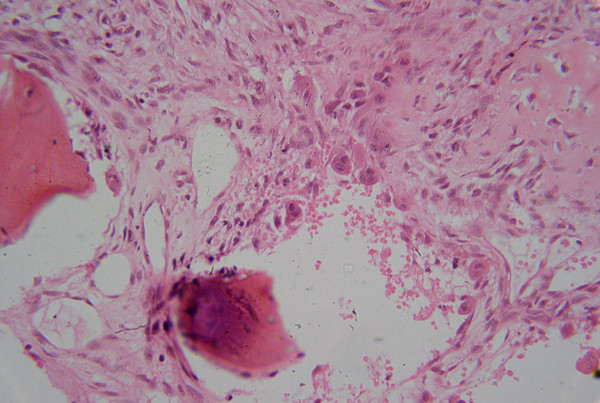
Microphotograph showing vascular spaces separated by septa containing giant cells and fibroblasts. (H&E 250×)

Histopathological examination (Figure 3) shows numerous variably sized blood filled spaces separated by fibrous septae containing spindle shaped cells and scattered multinucleated giant cells.

## Discussion

ABC is a pseudocyst lacking true epithelial lining and usually affecting long bones (50%) and vertebrae (20%) [3]. In facial skeleton, its occurrence is rare with mandible being affected more than maxilla (3:1) affecting mainly molar regions [1,2]. It accounts for 1.5% of the nonodontogenic, nonepithelial cysts of the mandible [9]. The mean age of involvement in skull and facial region is reported to be 14.3 years [10] with no sex predilection [1].

It is being recognized since 1893, but in 1942 Jaffe and Lichtenstein coined the term *aneurysmal cyst *which was changed to *aneurysmal bone cyst *in 1950 [3]. It is an expansile osteolytic pseudocyst which can attain great dimensions and may cause symptoms owing to its site and size and rapidity of growth i.e. swelling, deformity, pain, neurologic symptoms, and pathologic fractures.

Aneurysmal bone cyst is most common in those regions of the skeleton where there is both a relatively high venous pressure and high marrow content [11]. This explains rarity of ABC in the skull bones in which there is low venous pressure. According to WHO, ABC is an "expanding osteolytic lesion consisting of blood-filled spaces of variable size separated by connective tissue septa containing trabeculae of osteoid tissue and osteoclast giant cells."[12] Etiopathogenetically, it is thought to be a neoplasm, a developmental anomaly, or a response to trauma, chronic infection, arteriovenous anomalies or degenerative lesion [2,13,14].

It can occur as a primary lesion or secondarily in a preexisting lesion. Martinez V and Sissons HA concluded that most of these cysts occur as a primary lesion [15]. Giant cell tumor is the most common lesion associated with secondary ABC accounting for 39% of these lesions and similarly in 14% cases of giant cell tumor, ABC components are seen. Murphey MD et al has detailed radiopathologic correlations of ABC and giant cell tumor [16]. The other associated lesions are unicameral cyst, nonossifying fibroma, osteoblastoma, hemangioma, histiosarcoma, hemangioendothelioma, fractures and trauma [2].

In our case, there was no history or evidence of any other associated lesion. So we label it a case of primary ABC of coronoid process of mandible.

There are various treatment options suggested in the literature ranging from percutaneous sclerotherapy, diagnostic and therapeutic embolization, curettage, block resection and reconstruction, radiotherapy and systemic calcitonin therapy. Self healing cases have also been reported on long term follow up [17].

Dubois et al [18] reported good results with sclerotherapy, but there is no material available for histological confirmation and diagnosis is solely bases on clinical and radiological evidences in many cases in his series. He considered histological confirmation not mandatory for the diagnosis. It may miss many associated lesions. Furthermore he considered a copious blood return through the first puncture a necessary requisite for diagnosis; it may misdiagnose a case like ours where only brownish serous fluid came out from the cyst.

Embolization of the feeding vessels has been shown to be effective as a preoperative procedure to reduce peroperative bleeding or after surgical failure. It is also employed as a sole treatment modality. But ABC frequently lacks large feeding vessels therefore may require repeated sittings for embolization [19].

Radiation was not considered for this patient looking at his age and surgically accessible tumor.

We chose superior approach to infratemporal fossa as it had some distinct advantages in this case. We could approach temporal, infratemporal regions as well as origin of the ABC with single incision. There were minimum chances of injury to facial nerve. With this incision, we could approach infratemporal fossa and coronoid process from laterally also in case of need.

There was no excessive bleeding from the cyst in our case.

## Conclusion

- We presented first reported case of primary ABC from coronoid process of the mandible presenting as temporal swelling.

- Superior approach to infratemporal fossa is a reasonable approach while managing such cases providing wide exposure and access to almost all parts of the lesion with flexibility of surgical approach in case of need with the same incision.

- We conclude that even after relatively smaller area of bone involvement and without surrounding destruction; ABC of coronoid process can attain enormous dimensions to present as swelling of a distant region which is more important in relatively compact maxillofacial region with regards to symptoms and management.

## Competing interests

The author(s) declare that they have no competing interests.

## Authors' contributions

All five authors

1) Have made substantial contributions in management of this case and in conception, design, analysis and interpretation of results of this case report

2) Have been involved in drafting the article or revising it critically for important intellectual content.

3) Have given final approval to the version to be published

## Pre-publication history

The pre-publication history for this paper can be accessed here:


